# Supplementary material for: MUC5B rs35705950 Promoter Variant Is Associated with Usual Interstitial Pneumonia in Patients with Antisynthetase Syndrome
Source: J Clin Med. 2024 Oct 16;13(20):6159. doi: 10.3390/jcm13206159 (PMC11508769; doi:10.3390/jcm13206159)
Supplement: Supplementary file 1 [file jcm-13-06159-s001.zip › jcm-3169090-supplementary.pdf]

Supplementary material

Suplementary Table S1.

Sociodemographic, tomographic, and respiratory functional test characteristics according to genotype in patients with antisynthetase syndrome.

Onset characteristics	GG	GT	TT
	n =44	n = 15	n=1
**Demographics**			
Age, years	56.9 ± 10.3	61.5 ± 11.3	57
Sex, female§	32 (72.7)	10 (66)	1(100)
**Tobacco smoking history§**	14 (31.8)	4 (26.6)	0
**Time from respiratory symptoms to ILD diagnosis, months**	5.5 [2-12]	12 [3-25]	12
**HRTC pattern§**			
NSIP/OP	40(91)	10 (67)	1(100%)
UIP	4 (9)	5 (33)	0
**Antibody subtype§**			
Anti-Jo1	12 (27.0)	4 (26.6)	0
Non-anti-Jo1 (others)			
Anti-PL7	13 (29.5)	2 (13.3)	1 (100%)
Anti-PL12	10 (22.7)	7 (46.6)	0
Anti-EJ	7 (15.9)	1 (6.66)	0
Anti-OJ	2 (4.54)	1 (6.66)	0
**Pulmonary function tests**			
FVC (%)	63.7 ± 23.9	70.2 ± 27.9	29.0
DL_{CO} (%)	52.8 ± 27.3	65.8 ± 26.2	23.0

Notes: Data are presented as numbers (percentages); otherwise, they are given as the mean ± standard deviation or median [interquartile range]. ILD: interstitial lung disease, ASSD: anti-synthetase syndrome, NSIP: non-specific nonspecific interstitial pneumonia, UIP: usual interstitial pneumonia.
